# Histone-Like Nucleoid Structuring Protein Modulates the Fitness of *tet*(X4)-Bearing IncX1 Plasmids in Gram-Negative Bacteria

**DOI:** 10.3389/fmicb.2021.763288

**Published:** 2021-11-11

**Authors:** Wenhui Cai, Feifei Tang, Lijie Jiang, Ruichao Li, Zhiqiang Wang, Yuan Liu

**Affiliations:** ^1^College of Veterinary Medicine, Yangzhou University, Yangzhou, China; ^2^Jiangsu Co-innovation Center for Prevention and Control of Important Animal Infectious Diseases and Zoonoses, Yangzhou University, Yangzhou, China; ^3^Joint International Research Laboratory of Agriculture and Agri-Product Safety, The Ministry of Education of China, Yangzhou University, Yangzhou, China; ^4^Institute of Comparative Medicine, Yangzhou University, Yangzhou, China

**Keywords:** H-NS protein, fitness, *tet*(X4), IncX1 plasmids, Gram-negative bacteria (GNB)

## Abstract

The emergence of plasmid-mediated tigecycline resistance gene *tet*(X4) poses a challenging threat to public health. Based on the analysis of *tet*(X4)-positive plasmids in the NCBI database, we found that the IncX1-type plasmid is one of the most common vectors for spreading *tet*(X4) gene, but the mechanisms by which these plasmids adapt to host bacteria and maintain the persistence of antibiotic resistance genes (ARGs) remain unclear. Herein, we investigated the underlying mechanisms of how host bacteria modulate the fitness cost of IncX1 plasmids carrying *tet*(X4) gene. Interestingly, we found that the *tet*(X4)-bearing IncX1 plasmids encoding H-NS protein imposed low or no fitness cost in *Escherichia coli* and *Klebsiella pneumoniae*; instead, they partially promoted the virulence and biofilm formation in host bacteria. Regression analysis revealed that the expression of *hns* gene in plasmids was positively linked to the relative fitness of host bacteria. Furthermore, when pCE2::*hns* was introduced, the fitness of *tet*(X4)-positive IncX1 plasmid pRF55-1 without *hns* gene was significantly improved, indicating that *hns* mediates the improvement of fitness. Finally, we showed that the expression of *hns* gene is negatively correlated with the expression of *tet*(X4) gene, suggesting that the regulatory effect of H-NS on adaptability may be attributed to its inhibitory effect on the expression of ARGs. Together, our findings suggest the important role of plasmid-encoded H-NS protein in modulating the fitness of *tet*(X4)-bearing IncX1 plasmids, which shed new insight into the dissemination of *tet*(X4) gene in a biological environment.

## Introduction

Antibiotic resistance has constituted a growing threat to global public health. According to the data released by WHO, it is estimated that by 2050, the number of human deaths caused by multiple-drug resistance will increase to 10 million, surpassing the number of cancer deaths and becoming one of the leading causes of human death worldwide (Kuehn, 2011). Because of the widespread distribution of carbapenem resistance gene, *bla*_NDM_ (Nordmann et al., 2011), and polymyxin resistance gene, *mcr-1* (Liu et al., 2016), worldwide, the clinical efficacy of carbapenems and polymyxin, two high-priority agents for treatment of multidrug-resistant (MDR) Gram-negative bacterial infections (Kumarasamy et al., 2010; Rodríguez-Baño et al., 2018), was severely diminished. Therefore, tigecycline is recognized as the last option for treating serious infections (Tasina et al., 2011). However, with the emergence of high-level tigecycline resistance gene *tet*(X3/X4) (He et al., 2019; Sun et al., 2019), few choices were left for clinicians from the traditional antibiotic pipeline. Conjugable plasmid-mediated horizontal gene transfer is the dominant pathway accounting for the spread of antibiotic resistance genes (ARGs) (San Millan, 2018; Lerminiaux and Cameron, 2019). Plasmids, as mobile genetic elements, mediate the transmission of genetic information between bacteria and promote the adaptation of bacteria to various environments and the development of bacterial resistance (Lopatkin et al., 2017). Nevertheless, the high expression of plasmid-related genes would impose additional energy and metabolic burden to host bacteria, thereby resulting in fitness cost (San Millan and MacLean, 2017). Accordingly, the fitness cost elicited by resistance plasmids is closely associated with the dissemination of ARGs (Alonso-Del Valle et al., 2021). Since its first description in China in 2019, *tet*(X4)-bearing plasmids have been found worldwide (Ding et al., 2020; Zhang et al., 2020; Mohsin et al., 2021). For example, it is reported that *tet*(X4)-positive plasmids were widely distributed in the gut microbiota of Singaporeans (Ding et al., 2020). Besides, *tet*(X4)-bearing plasmids were identified from poultry, food, and environmental samples in South Asia (Mohsin et al., 2021). The prevalence of *tet*(X4) gene has posed a great challenge to human safety (Fu et al., 2021). IncX1 plasmid is one of the important vectors of *tet*(X4) gene (Li et al., 2020b), but the physiological mechanisms of widespread of *tet*(X4)-harboring IncX1 plasmids in different host strains are still unclear.

Histone-like nucleoid structuring (H-NS) protein is a kind of DNA-binding protein, which exists widely in Gram-negative bacteria (Navarre et al., 2006). H-NS protein not only participates in the regulation of cell metabolism and cellular stress response, but also responds to various environmental changes, such as pH, metal ion concentration, osmotic pressure, temperature, and stringent response (Gao et al., 2018). Notably, H-NS protein can act as a transcriptional inhibitor, silencing the expression of many genes (Dorman, 2007), including pathogenicity islands (SPIs) and fimbriae gene *pef* in *Salmonella* (Ali et al., 2014; Hurtado-Escobar et al., 2019), virulence genes *virF*, *virB*, and *icsA/B/P* in *Shigella* (Picker and Wing, 2016) and some costly conjugation genes (Doyle et al., 2007). The basis of H-NS silencing gene is its ability to target an AT-rich sequence, thus hindering its transcription (Dorman, 2014). As a global regulator, it is indicated that H-NS can regulate bacteria fitness by inhibiting the expression of certain genes. For example, the overexpression of the *Salmonella* pathogenicity island SPI2 generally acquired by horizontal gene transfer can lead to the growth defect of *Salmonella*, and H-NS inhibited the expression of SPI2 to improve the fitness of *Salmonella* (Lucchini et al., 2006). It was found that some plasmids also have genes encoding H-NS family proteins, and the deletion of *hns* decreases the fitness of host bacteria (Doyle et al., 2007), indicating that H-NS is likely to mediate the fitness cost of plasmids. A recent study showed that H-NS plays an important role in the dissemination of *bla*_NDM–__1_-bearing IncX3 Plasmid in *Escherichia coli* by regulating the expression of plasmid-related genes (Liu et al., 2020).

In this study, we explored the role of H-NS protein in the prevalence of *tet*(X4)-bearing IncX1 plasmids by a series of assays, including bacterial growth curve, relative fitness evaluation, and virulence assay. Most importantly, we investigated the correlation between the expression of hns and fitness cost of *tet*(X4)-positive IncX1 plasmids in various host bacteria. Our data show that H-NS plays a crucial role in alleviating the fitness cost of plasmids, which may explain the successful epidemic of *tet*(X4)-positive IncX1 plasmids in the clinical setting and provide new insights for addressing the global bacterial resistance crisis.

## Materials and Methods

### Bacterial Strains and Plasmids

The accession numbers of 51 *tet*(X4)-positive plasmids from NCBI databases are listed in Supplementary Table 1. Three *tet*(X4)-positive plasmids carrying *hns* gene used in this experiment are shown in Supplementary Table 2 and Supplementary Figure 1, in which pRF14-1 plasmid was isolated from the slaughterhouse of Jiangsu Province, China (Li et al., 2020b), and the other two plasmids pSC4R and pHS14-2 were isolated from Chinese pork samples. Standard strains involving *E. coli* TOP10, *Klebsiella pneumoniae* ATCC700603, and *Salmonella* Enteritidis ATCC13076 were used in this experiment.

### Plasmid Extraction, Whole Genome Sequencing, and Bioinformatics Analysis

Plasmids were extracted using the Plasmid Mini Kit I (Omega, China). Combined with the highly accurate short-read Illumina and long-read Oxford Nanopore Technologies (ONT) MinION platforms, WGS and *de novo* assembly using the hybrid strategy was performed. Plasmid sequences were annotated using the RAST^1^ automatically and modified manually. BRIG (Alikhan et al., 2011) and Easyfig (Sullivan et al., 2011) tools were used to visualize the genetic comparisons.

### Preparation of Competent Cells and Electroporation Experiment

As described in a previous study (Choi et al., 2006), the *E. coli* TOP10, *K. pneumonia*e ATCC700603, and *S.* Enteritidis ATCC13076 were cultured in LB broth to the logarithmic growth phase at 37°C and 200 rpm. The strains were precooled for 30 min and centrifuged at 4°C and 5,000 rpm for 5 min. Then, the clumps of bacteria are washed with water once and with 10% glycerin three times. Bacterial liquid (50 ml) was condensed into three tubes of 100 μl of competent cells.

A tube of competent cells was taken and then mixed with the plasmids including pRF14-1, pSC4R, and pHS14-2 separately (the volume is not greater than 10% of the competent cells volume). Immediately after electroporation, 1 ml of LB was added to the competent cells and recovered in 37°C and 200 rpm for 1 h. Then, the positive strains were screened by agar plate with tigecycline (2 μg/ml) and polymerase chain reaction (PCR) was used to verify whether the plasmid was successfully transferred into the cell. Nine transformants were obtained.

### Bacterial Growth Curve Assay

According to a previous method (Liu et al., 2020), overnight cultures of strains carrying pRF14-1, pSC4R, pHS14-2, and its recipient bacteria were diluted 1:1,000 into fresh LB broth and were incubated at 37°C with 200 rpm for 12 h. Growth curves were obtained by measuring the optical density of cultures at 620 nm every hour by Multiskan FC (Thermo Fisher Scientific). All experiments were conducted in triplicate and repeated three times independently, and the average values were used to estimate growth parameters.

### Plasmid Stability Experiment

Plasmid stability testing was performed by the serial passage method for 25 consecutive days at 1:1,000 dilutions without antibiotic pressure (two generations of growth per day) (Gao et al., 2020). Strains with different plasmids were propagated in antibiotic-free LB medium at 37°C with 200 rpm and shaken for 25 days (50 generations) to determine their stability in a different strain background. PCR was conducted every 10 generations to detect the plasmid and *tet*(X4).

### *In vitro* Competition Experiment

Overnight cultures of plasmid-carrying clone and corresponding recipient bacteria were diluted 1:1,000 in LB broth and mixed at 1:1 ratio. Then, this mixture was incubated for 3 days at 37°C with 200 rpm, and diluted 1:100 into fresh LB broth every 24 h. The competition mixture at 0, 24, 48, and 72 h was plated on LB agar without drug and LB agar containing tigecycline with proper dilution to count the colony numbers. The formula Wt = ln(N_f,__R_/N_i,__R_)/ln (N_f, S_/N_i, S_) was used to calculate the Wt, namely, relative fitness of plasmid-carrying strain (R) compared to the recipient strain (S). N_i,__R_ and N_f,__R_ are the numbers of cells of R at the beginning and end of the competition, and N_i__, S_ and N_f__, S_ are the densities of cells of S at the start and end of the competition, respectively (DelaFuente et al., 2020). Each experiment was set in three parallel and repeated three times.

### Biofilm Formation Assay

Biofilm formation assay was performed using crystal violet staining (Rossi Gonçalves et al., 2017). Specifically, 200 μl of bacteria suspension containing 1 × 10^6^ CFU/ml prepared in LB broth was added to 96-well polystyrene plates (flat bottom with cover, aseptic) and incubated at 37°C for 36 h, at which time the biofilm had been attached to the bottom and wall of the well, and the culture medium was carefully removed. The wells were washed with PBS twice and fixed 15 min with methanol of 50 μl. The methanol was removed and the 0.1% crystal purple solution of 100 μl was added to stain 30 min. After cleaning the wells with PBS, 33% glacial acetic acid solution of 100 μl was added, and 30 min was incubated at 37°C. The OD value at 570 nm of each well was determined. The same operation was carried out with the LB broth without bacteria as a negative control. Experiments were performed with three biological replicates.

### *Galleria mellonella* Infection Model

In order to evaluate the effects of three plasmids on the virulence of different receptor bacteria, *G. mellonella* larvae were used as an *in vivo* infection model (Tsai et al., 2016). Eight healthy and uniform larvae were used for each strain. The bacterial particles were washed with aseptic saline and then they were diluted with aseptic saline to an appropriate concentration. Using the Hamilton syringe of 25 μl, the bacterial liquid of 10 μl was inoculated into the left forefoot of the last pair of larvae. There were two control groups; one was inoculated with 10 μl of sterile saline, and the other received simulated injection to ensure no physical trauma. All the larvae were incubated in a constant temperature incubator at 37°C for 5 days. During this period, the larvae were observed. If the larvae no longer move when they are touched or blacken, they are considered dead. Results were analyzed by Kaplan–Meier survival curves (GraphPad Prism version 8.3.0 statistics software).

### Motility Test

The method of semisolid medium was used to determine the movement ability of bacteria (Liu et al., 2020). Several single colonies were picked and were cultured in LB broth. Then, the bacteria liquid was centrifuged and resuspended with sterile saline. Meanwhile, sterile saline was used to uniformly adjust the concentration of bacteria to 0.6 McFarland. Then, 0.3% semisolid medium (3 g/L) agar plate was prepared, and the 2 μl of bacterial solution was added to the center of the semisolid medium. After the plates were incubated at 37°C for 48 h, the size of bacterial colonies was measured.

### Quantitative Real-Time PCR

The absolute expression of *hns* and *tet*(X4) was detected by RT-qPCR. Firstly, *hns* and *tet*(X4) genes were, respectively, cloned into pBAD and pCE2 to generate pBAD-*hns* and pCE2-*tet*(X4). Plasmid pBAD-*hns* and pCE2-*tet*(X4) were used as template DNA with primers *hns*-RNA-F, *hns*-RNA-R, *tet*(X4)-RNA-F, and *tet*(X4)-RNA-R, respectively, to set up the corresponding standard curve. The total RNA of bacteria was extracted by Bacteria RNA Extraction Kit (Vazyme Biotech Co., Ltd) and then reverse transcribed into cDNA by 1 μg of RNA using the HiScriptR^R^ III RT SuperMix for qPCR (+gDNA wiper) Kits (Vazyme Biotech Co., Ltd), and the cDNA of 1 μl was used as template for RT-qPCR with primers *hns*-RNA-F, *hns*-RNA-R, *tet*(X4)-RNA-F, and *tet*(X4)-RNA-R using the ChamQTM Universal SYBRR^R^ Color qPCR Master Mix Kits (Vazyme Biotech Co., Ltd). According to the standard curve, the absolute expression of *hns* and *tet*(X4) genes was calculated. The specific sequence of the primers is shown in Supplementary Table 3.

### Determination of Uronic Acid Content

The strains in the exponential growth phase 400 μl were harvested by centrifugation at 8,000 × *g* for 10 min at 4°C and washed with 500 μl of 1 × PBS three times. Next, the bacteria were resuspended in 500 μl of Tris-HCl buffer (pH = 7.0) and centrifuged at 8,000 × *g* for 5 min at 4°C to remove the supernatant and then resuspended with 500 μl of Tris-HCl buffer again. Subsequently, 2.4 ml of 12.5 mM tetraborate solution was mixed and heated at 100°C for 5 min. All of the samples were placed in ice, and 40 μl of 15% *m*-hydroxybiphenyl solution was added to each sample. Thorough mixing was needed. Finally, the concentration of each sample was detected in UV-visible spectroscopy reader at 520 nm (Zheng et al., 2020).

### Statistical Analysis

Data are expressed as mean ± standard deviations (SD), and analyzed by either a non-parametric one-way ANOVA, unpaired *t-*test, or two-way ANOVA. Statistical analysis was conducted using GraphPad Prism version 8.3.0 statistics software. Results were considered significant with *p* < 0.05.

## Results

### The Distribution of *hns* in IncX1-Type *tet*(X4)-Positive Plasmids

**FIGURE 1:**
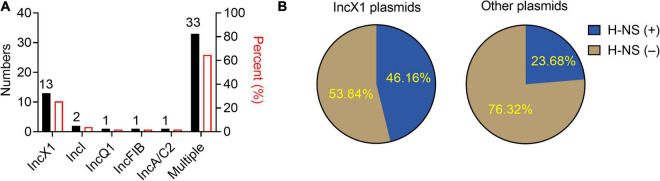
IncX1 type is dominant in *tet*(X4)-bearing plasmids encoding H-NS from NCBI database. **(A)** The histogram shows plasmid typing and proportion of *tet*(X4)-positive plasmids from NCBI database. The black columns represent the numbers of *tet*(X4)-positive plasmids in different types. The red columns represent the corresponding percentage. **(B)** The left side of the pie graph shows the carrying rate of *hns* in *tet*(X4)-positive IncX1 plasmids. On the right is the carrying rate of *hns* gene in other types of plasmids.

To understand the epidemic characteristics of *tet*(X4)-positive plasmids, the replicon types of a total of 51 *tet*(X4)-positive plasmids from different bacteria in NCBI database were analyzed. We found that the plasmids with multiple replicon types were the most common in *tet*(X4)-positive plasmids, while IncX1 plasmids were dominant in single replicon plasmid types, accounting for 72.2% (Figure 1A). Then, the genome sequences of these IncX1 plasmids were analyzed, and it was found that nearly half of the plasmids carry *hns* gene (46.16%) (Figure 1B). Combining these results, we hypothesized that H-NS protein may play an unappreciated role in the widespread prevalence of *tet*(X4)-harboring IncX1 plasmids.

### Analysis of Fitness of *tet*(X4)-Positive Plasmids Encoding Histone-Like Nucleoid Structuring Protein

**FIGURE 2:**
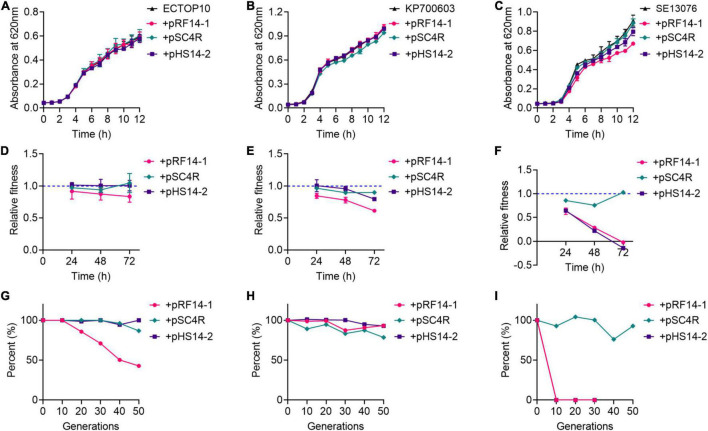
*tet*(X4)-positive IncX1 plasmids encoding H-NS protein displays low fitness cost in host bacteria. **(A–C)** Growth curves of transformants with *E. coli* TOP10 **(A)**, *K. pneumoniae* ATCC700603 **(B)**, or *S.* Enteritidis ATCC13076 **(C)** as recipient strains. **(D–F)** Relative fitness of transformants with *E. coli* TOP10 **(D)**, *K. pneumoniae* ATCC 700603 **(E)**, or *S.* Enteritidis ATCC13076 **(F)** as recipient strains at 24, 48, and 72 h. **(G–I)** Plasmid stability in three recipient strains, including *E. coli* TOP10 **(G)**, *K. pneumonia*e ATCC700603 **(H)**, or *S.* Enteritidis ATCC13076 **(I)**. Data are representative of three biological replicates and shown as mean ± SD.

Three *hns* and *tet*(X4)-positive IncX1 plasmids, including pRF14-1, pSC4R, and pRF14-1, were applied as models to explore the role of H-NS protein in the prevalence of *tet*(X4) gene. Considering that fitness cost elicited by resistance plasmids directly affects the dissemination of resistance genes, we hypothesize that H-NS protein may modulate the fitness of host bacteria, which is critical for the transmission of plasmids. To test this hypothesis, these three plasmids were transferred into three recipient bacteria involving *E. coli* TOP10 (abbreviated as ECTOP10), *K. pneumoniae* ATCC700603 (abbreviated as KP700603), and *S.* Enteritidis ATCC13076 (abbreviated as SE13076) separately, and a total of nine transformants were obtained and designated as ECTOP10/pRF14-1, ECTOP10/pSC4R, ECTOP10/pHS14-2, KP700603/pRF14-1, KP700603/pSC4R, KP700603/pHS14-1, SE13076/pRF14-1, SE13076/pSC4R, and SE13076/pHS14-2, respectively. We analyzed the fitness of these nine transformants and their corresponding recipient bacteria, and determined their growth curve, relative fitness, and plasmid stability. Except for the slight slow growth of SE13076/pRF14-1, there was no significant difference in the growth curve in other transformants compared with recipient bacteria (Figures 2A–C). These results showed that the three *tet*(X4)-positive IncX1 plasmids encoding H-NS protein did not affect the growth rate of host bacteria, implying that the acquisition of these plasmids has no antagonistic effect on the growth of host cells.

Next, the *in vitro* competitive test, the most direct method for fitness evaluation (DelaFuente et al., 2020), was used to assess the fitness cost of three plasmids in different hosts. In ECTOP10 and KP700603, these three plasmids did not show obvious fitness cost at both three time points (Figures 2D,E), which were in agreement with the results of growth kinetics. It is demonstrated that the fitness cost is the key factor affecting the replacement rate of drug-resistant bacteria by sensitive bacteria (De Gelder et al., 2004). These results implied that the prevalence of *tet*(X4)-positive IncX1 plasmids may be due to their small fitness cost on host bacteria. Moreover, we found that even without antibiotic pressure, these three plasmids still conferred a fitness advantage in ECTOP10 during 40 passages (Supplementary Figure 2). In contrast, in SE13076 host bacteria, plasmids pRF14-1 and pHS14-2 showed a high fitness cost, especially after 24 h, whereas plasmid pSC4R did not show obvious fitness cost (Figure 2F).

The high cost of resistance plasmids may also be manifested by the instability of plasmids in host bacteria (Wu et al., 2021). To fully understand the dissemination of *tet*(X4)-positive IncX1 plasmids, we investigated the stability of these plasmids in three host bacteria. The strains carrying plasmids were passaged for 25 days (a total of 50 generations) in the absence of drugs, and then the loss rate of plasmids at every 10 generations was detected. We found that plasmids pSC4R and pHS14-2 showed good stability in both ECTOP10 and KP7000603. The plasmid pRF14-1 had good stability in KP700603, but in ECTOP10, it began to be lost at the 10th generation, and the plasmid loss rate reached about 60% by the 50th generation (Figures 2G,H). For SE13076, the plasmid pSC4R showed good stability, while the other two plasmids were unstable and completely lost in the 10th generation (Figure 2I). The instability of these two plasmids in SE13076 may due to the low compatibility between plasmids and host bacteria. Thus, the high fitness cost of pRF14-1 and pHS14-2 in SE13076 may be attributed to their poor stability. Further conjugation experiments indicated that plasmid pRF14-1 had the highest conjugation frequency (Supplementary Figure 3), which may partly affect its stability in the recipient. Consistently, a previous study has shown that conjugation can easily offset the fitness cost of plasmids (Lopatkin et al., 2017), especially the high conjugation frequency. Combining with *in vitro* competition experiments and growth kinetics, these results indicate that *tet*(X4)-positive IncX1 plasmids carrying *hns* gene display great fitness advantages in various host bacteria.

### Effect of *tet*(X4)-Positive Plasmids on Virulence of Host Bacteria

**FIGURE 3:**
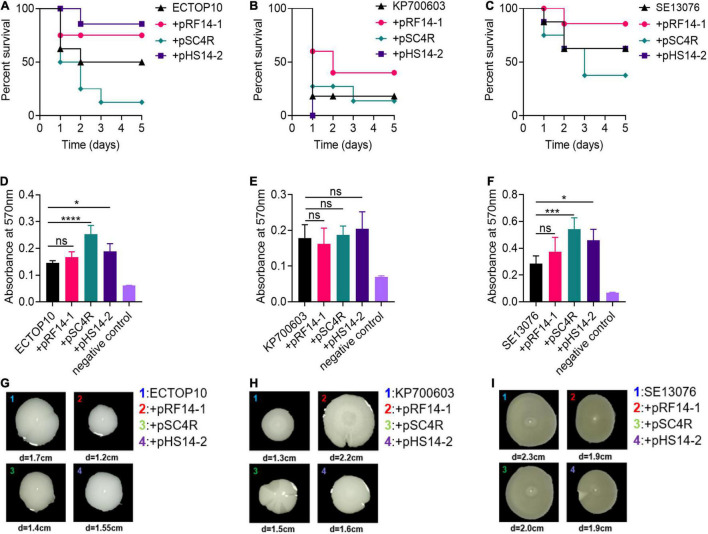
Effect of *tet*(X4)-bearing IncX1 plasmids encoding H-NS on the virulence phenotype of different recipient strains. **(A–C)** Survival of *G. mellonella* larvae infected by recipient strains and corresponding transformants, including *E. coli* TOP10 **(A)**, *K. pneumonia*e ATCC700603 **(B)**, or *S.* Enteritidis ATCC13076 **(C)**. **(D–F)** Biofilm formation ability of three recipient strains and corresponding transformants, including *E. coli* TOP10 **(D)**, *K. pneumoniae* ATCC700603 **(E)**, or *S.* Enteritidis ATCC13076 **(F)**. Biofilm mass was monitored using crystal violet staining. Data are representative of three biological replicates and shown as mean ± SD. *p*-values were determined using a non-parametric one-way ANOVA (*p < 0.05; ***p < 0.001; ****p < 0.0001; ns, not significant). **(G–I)** Swimming motility of three recipient strains and corresponding transformants. The mean diameters from three biological replicates were shown.

It is suggested that the fitness cost of antibiotic resistance plasmids may result in virulence reduction of host bacteria (Yang et al., 2017). To analyze the impact of *tet*(X4)-positive plasmids on the virulence of host cells, we first used the *Galleria mellonella* infection model to evaluate the pathogenicity changes of host bacteria after obtaining resistance plasmids. Interestingly, results showed that the carriage of plasmid pSC4R remarkably enhanced the virulence of host bacteria, while plasmid pRF14-1 reduced bacterial pathogenicity to larvae. However, the adoption of plasmid pHS14-2 showed different effect in three host bacteria (Figures 3A–C).

The formation of dormant or biofilm-grown cells has been one of the important ways for bacteria to adapt to the environmental pressure such as antibiotic killing (Flemming et al., 2016). Thus, we next investigated the effect of *tet*(X4)-positive IncX1 plasmids on the biofilm formation in host bacteria using crystal violet staining. These results showed that the introduction of plasmid pSC4R and pHS142 is able to significantly enhance the biofilm formation ability of ECTOP10 and SE13076, while plasmid pRF14-1 had no obvious effect on the biofilm generation in three host bacteria. In addition, the presence of three *tet*(X4)-positive plasmids did not influence the biofilm production in KP700603 (Figures 3D–F).

The swimming motility of bacteria is associated with the invasiveness and adhesion of pathogenic bacteria and thereby serves as an important indicator of bacterial virulence (Brunelle et al., 2017). Although the genes encoding bacterial flagella are usually on chromosomes (Samatey et al., 2001), the intake of plasmids will change the bacterial regulatory network (Li et al., 2020a), and the expression of plasmid genes will be strictly regulated accordingly. We compared the movement ability of bacteria with or without *tet*(X4)-positive IncX1 plasmids on 0.3% (w/v) agar media. In ECTOP10, plasmid pRF14-1 mildly reduced the movement ability of recipient bacteria, but markedly enhanced the swimming motility of KP700603 (Figures 3G,H). Considering that *K. pneumoniae* lack flagella, we supposed that the motility phenotype may be attributed to the capsule overproduction. Thus, we determined the capsule production of KP700603 and its transformants. Consistently, the results showed that plasmids pRF14-1 and pHS14-2 significantly increased the production of capsule in *K. pneumoniae* (Supplementary Figure 4). With regard to SE13076, these three plasmids had no remarkable effect on bacterial motility (Figure 3I). Collectively, our data suggest that the host bacteria commonly maintain their virulence, biofilm production, and motility after obtaining *tet*(X4)-positive plasmids carrying *hns* gene.

### Histone-Like Nucleoid Structuring Protein Reduces the Fitness Cost Elicited by *tet*(X4)-Positive Plasmids

**FIGURE 4:**
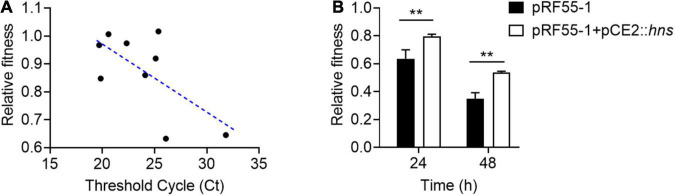
H-NS has a critical role in the fitness of *tet*(X4)-positive IncX1 plasmids. **(A)** Negative correlation between relative fitness and *hns* Ct value by quantitative real-time PCR. **(B)** The presence of H-NS protein improves the relative fitness of *E. coli* TOP10 carrying plasmid pRF55-1. Plasmid pRF55-1 is *tet*(X4)-bearing IncX1 plasmid without *hns* gene. Data are representative of three biological replicates and shown as mean ± SD. *p*-values were determined by unpaired *t-*test (**p < 0.01).

To understand whether H-NS really plays a direct role in plasmid fitness, we conducted a regression analysis of the relationship between the relative fitness of plasmid-bearing host bacteria and the expression of *hns* gene by RT-qPCR analysis. Ct values were used to represent the expression of *hns*; the smaller the Ct, the higher the expression of *hns* gene. Interestingly, we found that the Ct value of *hns* was negatively correlated with the relative fitness of *tet*(X4)-positive plasmids in host bacteria (Figure 4A), indicating that plasmid-encoded H-NS protein was positively linked with the relative fitness. To further verify this finding, we tried to construct the *hns*-deletion plasmid using both CRISPR-Cas9 or λRed homologous recombination. However, we failed to obtain the *hns*-deficient plasmid without changes in the plasmid backbone. We speculated that the presence of *hns* gene is critical for the stability of clinical *tet*(X4)-positive plasmids. To address this issue, we cloned *hns* gene into pCE2 vector and successfully constructed pCE2::*hns*. A *tet*(X4)-positive IncX1 plasmid pRF55-1 without *hns* gene was introduced into ECTOP10. Subsequently, we compared the fitness of ECTOP10/pRF55-1 transformant before and after the introduction of plasmid pCE2::*hns*. Consistent with the regression analysis, we found that the fitness of TOP10/pRF55-1 was markedly improved after the acquisition of plasmid pCE2::*hns* (Figure 4B). These data support our hypothesis that H-NS protein can regulate the fitness cost induced by *tet*(X4)-positive plasmids in bacteria.

### Fitness and *hns* Expression of Bacteria Carrying *tet*(X4)-Positive Plasmids Under Different Nutritional Conditions

**FIGURE 5:**
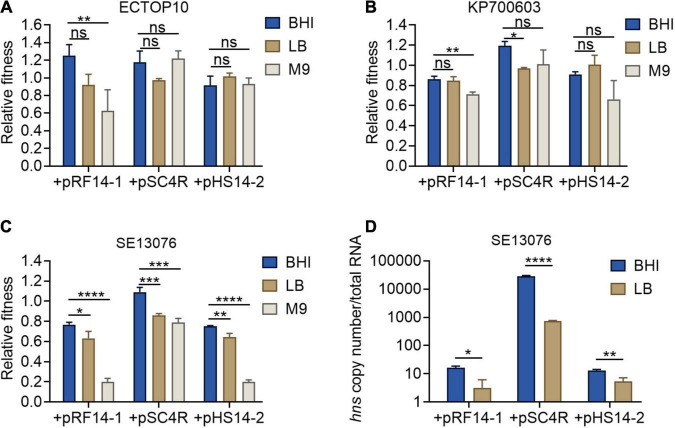
Fitness and *hns* expression of host bacteria under different nutritional conditions. **(A–C)** Relative fitness of transformants of *E. coli* TOP10 **(A)**, *K. pneumoniae* ATCC700603 **(B)**, or *S.* Enteritidis ATCC 13076 **(C)** carrying three *tet*(X4)-bearing IncX1 plasmids in high nutritional BHI culture, medium nutrition LB broth, and low nutrition M9 culture. *p*-values were determined using a non-parametric one-way ANOVA (*p < 0.05, **p < 0.01; ns, not significant). **(D)** Absolute expression of *hns* gene in transformants of *S.* Enteritidis ATCC13076 as recipient. Data are representative of three biological replicates and shown as mean ± SD. *p*-values were determined by unpaired *t-*test (*p < 0.05, **p < 0.01, ***p < 0.001, ****p < 0.0001; ns, not significant).

Previous studies have reported that the expression of *hns* gene is negatively correlated with the concentration of intracellular (p)ppGpp (Brandi et al., 2020). Thus, it is plausible that the accumulation of (p)ppGpp under the condition of nutrient deficiency would lead to the inhibition of *hns* expression. To further strengthen our findings on the relationship between *hns* expression and fitness of *tet*(X4)-positive plasmids, we determined the relative fitness of host bacteria carrying *tet*(X4)-positive plasmids under different nutritional conditions, including brain heart infusion (BHI) high nutrition broth, LB broth, and M9CA(M9) broth low nutrition medium. Consistently, host bacteria showed the highest fitness in BHI broth, but relatively low fitness in M9 medium (Figures 5A–C). In addition, we measured the expression of *hns* gene in BHI high nutrition broth and LB common broth using SE13076 carrying *tet*(X4)-positive plasmids as a representative strain. Due to the lack of nutrition in M9 medium, the bacteria could not be expanded in enough numbers for following RNA extraction. As expected, we found that the expression of *hns* gene in BHI broth was higher than that in LB broth (Figure 5D). Taken together, our findings reveal that the altered *hns* expression in host bacteria carrying *tet*(X4)-positive plasmids under different nutritional conditions is related to their fitness changes.

### Negative Correlation Between the Expression of *hns* and *tet*(X4) Gene

**FIGURE 6:**
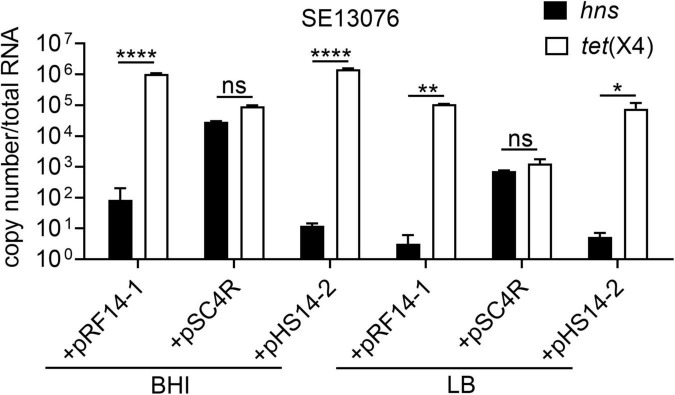
Negative correlation between absolute expression of *hns* and *tet(*X4) gene in *S.* Enteritidis ATCC 13076 transformants under different nutritional cultures. The copy number of *hns* and *tet*(X4) in transformants using *S.* Enteritidis ATCC13076 as a recipient strain in BHI, LB, and M9 media. Data are representative of three biological replicates and shown as mean ± SD. *p*-values were determined by two-way ANOVA (*p < 0.05; **p < 0.01; ****p < 0.0001; ns, not significant).

Having shown that plasmid-encoded H-NS protein plays an important role in the fitness of *tet*(X4)-bearing IncX1 plasmids, we next explored how H-NS regulates the adaptive cost of resistance plasmids in bacteria. To this end, we determined the correlation between the expression of *tet*(X4) and *hns* genes in SE13076 transformant. Interestingly, we found that the expression of *hns* in SE13076 carrying plasmids pRF14-1 or pHS14-2 was negatively correlated with that of *tet*(X4), while there was no significant relationship between *hns* and *tet*(X4) expresssion in plasmid pSC4R (Figure 6). In line with the previous relative fitness and stability results, the low relative fitness and instability of plasmids pRF14-1 or pHS14-2 in SE13076 may be attributed by the low expression of *hns* and the high expression of *tet*(X4) gene. By contrast, the better fitness advantage and stability of plasmid pSC4R may be caused by high expression of *hns* gene. Together, these results suggest that H-NS modulates fitness of *tet*(X4)-bearing IncX1 plasmids in Gram-negative bacteria by altering the expression of tigecycline resistance gene *tet*(X4).

## Discussion

The dissemination of *tet*(X4)-positive IncX1 plasmids have attracted much attention because it confers high level resistance to tigecycline, a clinically relevant antibiotic for Gram-negative bacterial infections (Ding et al., 2020; Li et al., 2020c). However, it remains unclear how *tet*(X4)-bearing IncX1 plasmids adapt to new hosts and further spread in various Gram-negative microorganisms. In this study, we investigated the potential role of plasmid-encoded H-NS protein in the dissemination of *tet*(X4)-bearing IncX1 plasmid by monitoring bacterial growth, fitness, stability, and virulence. Interestingly, we found that the carriage of *tet*(X4)-positive plasmids encoding H-NS had low fitness cost in *E. coli* TOP10 and *K. pneumoniae* ATCC700603, rather than in *S.* Enteritidis ATCC13076. These findings support the fact that *tet*(X4) was reported in *E. coli* and *K. pneumoniae*, but not in *S.* Enteritidis (He et al., 2019; Yu et al., 2021), suggesting that the *tet*(X4)-carrying plasmids impose a high cost in *Salmonella*. It would be interesting to investigate the underlying mechanisms behind this phenomenon. Furthermore, we found that the expression of *hns* gene was positively associated with the fitness advantages of *tet*(X4)-bearing IncX1 plasmids, indicating that H-NS plays a key role in the prevalence of *tet*(X4) genes in Gram-negative pathogens.

Accordingly, high-cost resistance plasmids will produce a burden in the host such as a reduced bacterial growth (San Millan and MacLean, 2017). For example, previous studies have reported that the high cost caused by overexpression of *mcr-1* can lead to bacterial growth defects (Yang et al., 2021) and the low-cost *bla*_NDM__–__5_-carrying IncX3 plasmid has no significant effect on the growth of bacteria (Ma et al., 2020). Consistently, our results showed that three *tet*(X4)-positive plasmids carrying *hns* gene did not bring obvious growth defects to the host bacteria, suggesting that these plasmids impose a low fitness cost. There were positive and negative effects between the acquisition of antibiotic resistance and bacterial virulence (Beceiro et al., 2013). The most worrying thing is that while antibiotic resistance is acquired, the virulence of bacteria is also increasing, which will greatly increase the difficulty of clinical treatment. In this study, plasmid pSC4R significantly enhanced the virulence of three recipient bacteria to larvae, which was consistent with the results of biofilm assay. Biofilms can adhere to cells and have an anti-phagocytic effect, thus enhancing the virulence of bacteria to the body (Koo et al., 2017). In fact, the phenotype of bacterial virulence is affected by many factors; in addition to the expression of virulence genes, it may also be affected by two-component regulatory systems, antibiotic resistance, and so on (Beceiro et al., 2013). For example, the expression of homogeneous methicillin resistance in *S. aureus* affects the biofilm phenotype and reduces virulence (Rudkin et al., 2012). Therefore, we supposed that plasmid pSC4R is likely to encode virulence regulators that enhance host virulence. The virulence difference of the same plasmid in various hosts implied that the virulence of bacteria may be regulated by both the host and plasmid, which requires further exploration.

It is suggested that the epidemic plasmids were stable in host bacteria, even in the absence of antibiotic pressure, and endow the host bacteria the advantage of fitness (Cottell et al., 2012; Fischer et al., 2014). Heretofore, some mechanisms have been shown to mitigate the fitness cost to host bacteria, such as compensatory mutations in host chromosomal genomes or plasmids. For example, the mutation of chromosome gene *gac*A/*gac*S, which is involved in the regulation of secondary metabolism, can alleviate the fitness cost of the pQBR103 plasmid in *Pseudomonas fluorescens* by reducing the expression of plasmid and chromosomal genes (Harrison et al., 2015). Another study showed that the *trf*A mutation in the plasmid pMS0506 leads to a significant increase in the copy number of plasmid, which greatly improved the plasmid stability and enhanced its adaptability to the host (Sota et al., 2010). In addition to compensatory mutations, there are also some regulatory factors that can directly or indirectly modulate the fitness of the plasmid-bearing host bacteria. For instance, it has been found that the plasmid pHNSHP45 carrying *mcr-1* resistance gene encodes a hypothetical ProQ/FinO family protein PcnR, which can control the appropriate expression of *mcr-1* by inhibiting the replication of plasmids, thereby reducing the fitness cost caused by high expression of *mcr-1* (Yang et al., 2021). The toxin–antitoxin (TA) system, which was originally found on plasmids, can also maintain the stable existence of plasmids (Hayes, 2003). Besides, several studies showed that H-NS protein can inhibit the expression of high-cost genes and improve the fitness advantages of bacteria (Ali et al., 2014; Jian et al., 2016). H-NS protein can bind to the gene promoter and further inhibit the transcriptional expression of genes (Navarre et al., 2006). Reducing the cost of plasmids by inhibiting the expression of high cost genes such as ARGs on *tet*(X4)-bearing IncX1 plasmids may be a key factor for the persistence of *tet*(X4)-plasmids in bacterial populations. This view was supported by our observation that plasmids pRF14-1 and pHS14-2 with low-level of *hns* gene expression had a lower fitness in SE13076. In addition, our study also proved that when the plasmid pCE2:*hns* was introduced, the fitness of plasmid pRF55-1 without *hns* in ECTOP10 was significantly improved, which clearly indicated that H-NS could improve the fitness of *tet*(X4)-bearing IncX1 plasmids. Consistent with our findings, it is shown that H-NS family proteins can regulate the expression of genes on the plasmid and further reduce the fitness cost of plasmids (Doyle et al., 2007). Nevertheless, the detailed mechanisms underlying how H-NS protein regulate the transcription and translation of *tet*(X4) gene warrant further studies.

## Conclusion

In conclusion, our study indicates that *tet*(X4)-bearing IncX1 plasmids carrying *hns* gene result in low fitness costs in various Gram-negative pathogens, but instead confer fitness advantages to the host such as enhanced virulence and biofilm formation. Importantly, we find that plasmid-encoded H-NS protein inhibits the expression of *tet*(X4) gene, thereby reducing the fitness cost of IncX1 plasmids. Together, these data highlight the important role of H-NS protein in the dissemination of *tet*(X4)-positive plasmids.

## Data Availability Statement

The original contributions presented in the study are included in the article/Supplementary Material, further inquiries can be directed to the corresponding author/s.

## Author Contributions

YL and ZW designed this study. WC and FT performed all experiments and wrote the draft manuscript. YL, WC, LJ, and RL analyzed the data. All authors have read and agreed to the published version of the manuscript.

## Conflict of Interest

The authors declare that the research was conducted in the absence of any commercial or financial relationships that could be construed as a potential conflict of interest.

## Publisher’s Note

All claims expressed in this article are solely those of the authors and do not necessarily represent those of their affiliated organizations, or those of the publisher, the editors and the reviewers. Any product that may be evaluated in this article, or claim that may be made by its manufacturer, is not guaranteed or endorsed by the publisher.

## Footnotes

1
http://rast.nmpdr.org/
