# Supplementary material for: Histone-Like Nucleoid Structuring Protein Modulates the Fitness of tet(X4)-Bearing IncX1 Plasmids in Gram-Negative Bacteria
Source: Front Microbiol. 2021 Nov 11;12:763288. doi: 10.3389/fmicb.2021.763288 (PMC8632487; doi:10.3389/fmicb.2021.763288)
Supplement: Supplementary file 1 [file Data_Sheet_1.docx]

**Supplementary Information**

**Figures**


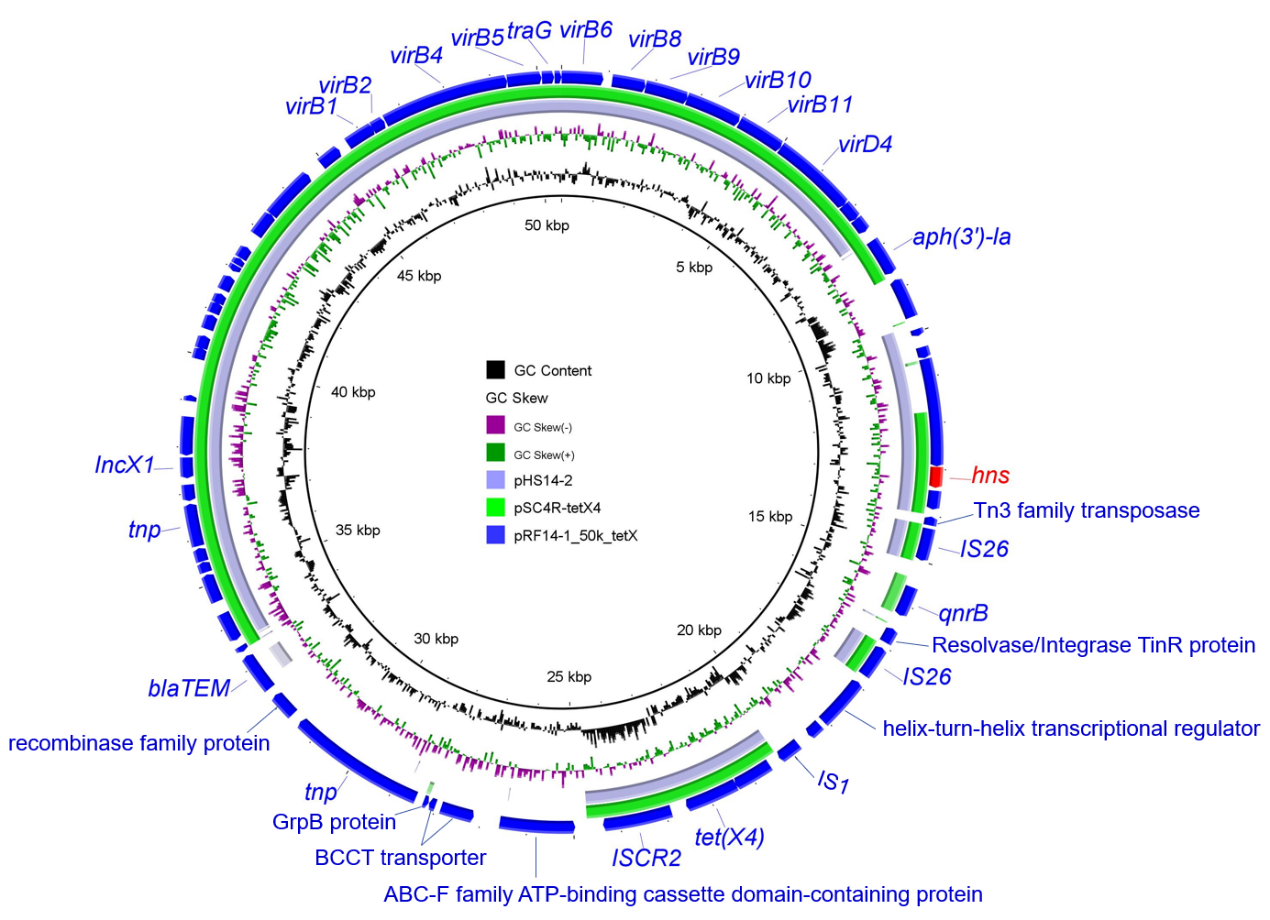


**Figure S1 Circular comparison between the *tet*(X4)-bearing IncX1 plasmid pRF14-1_50k_*tet*X and other IncX1 plasmids in the NCBI database.**

The *tet*(X4)-bearing IncX1 plasmid pRF14-1_50k_*tet*X was used as the reference in the outermost ring.

**
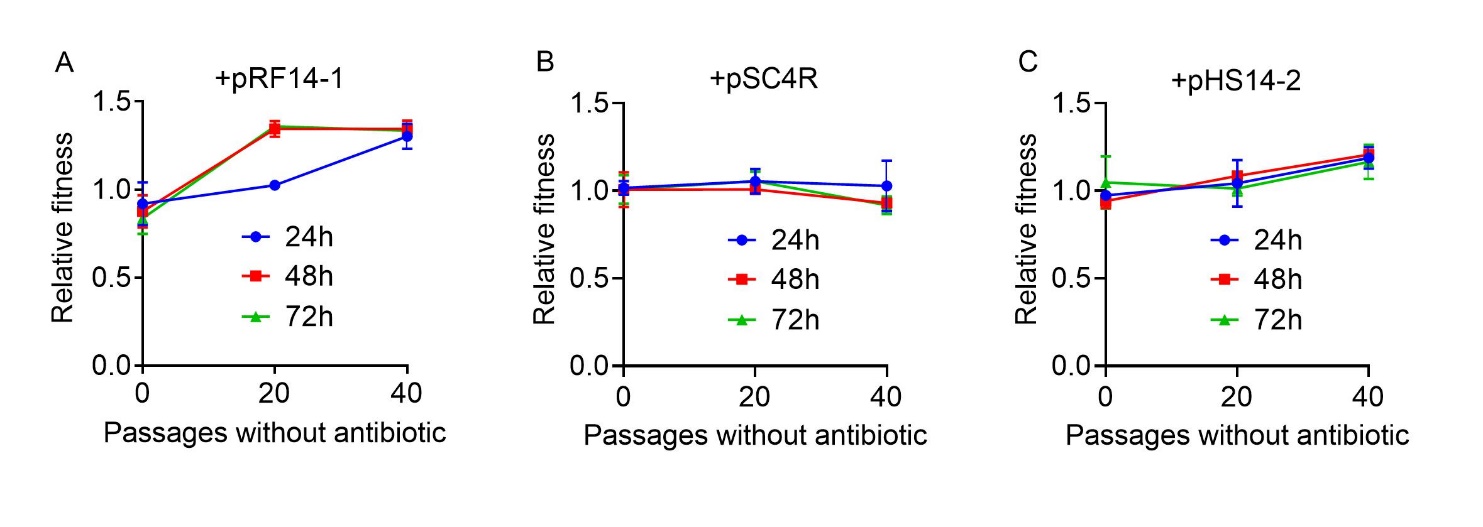
**

**Figure S2 Relative fitness of three *tet*(X4)-bearing IncX1 plasmids after passage in TOP10 host bacteria.**

Fitness analysis of the TOP10 carrying pRF14-1 **(A)**, pRSC4R **(B)** or pHS14-2 **(C)** during serial passaging in the absence of antibiotic. Data are representative of three biological replicates and shown as mean ± SD.


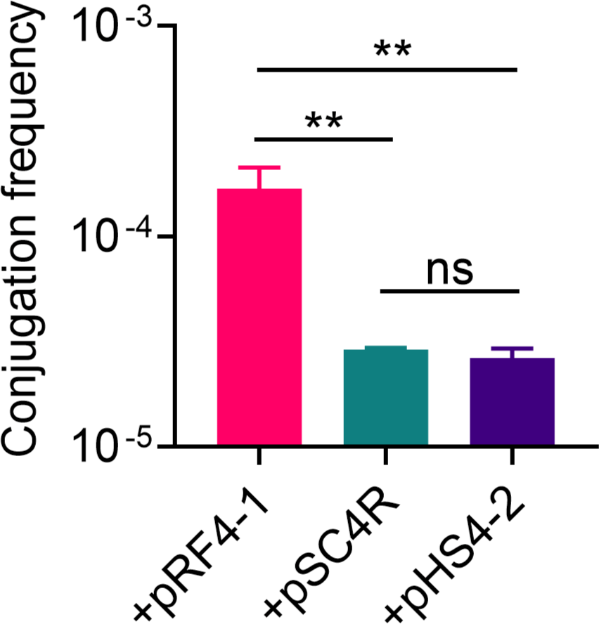


**Figure S3 Conjugation frequency of different *tet*(X4)-bearing IncX1 plasmids from *E. coli* TOP10 to *E. coli* C600.**

TOP10 is the donor bacteria and C600 is the recipient bacteria. Data are representative of three biological replicates and shown as mean ± SD.

**
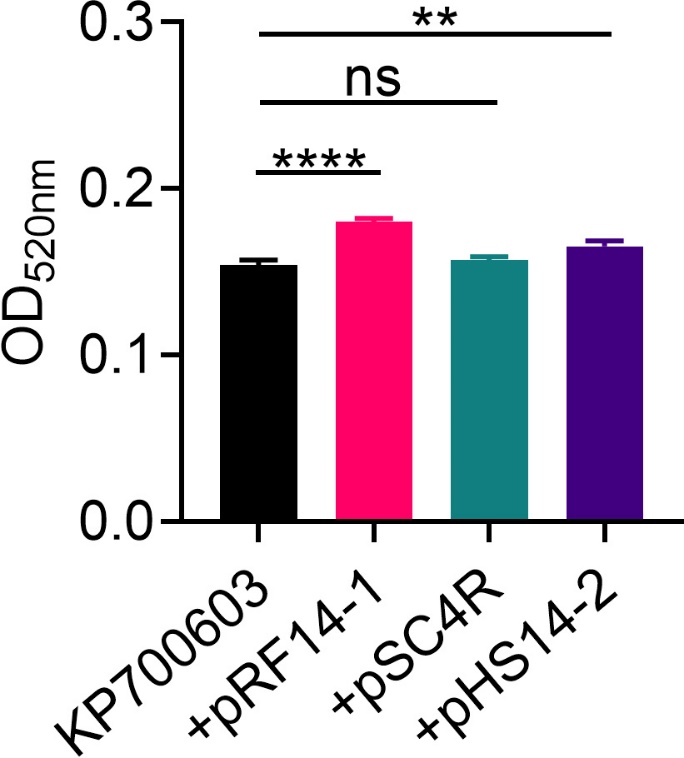
**

**Figure S4 Effects of different plasmids on capsule production of *K. pneumoniae*.**

Uronic acid contents in strains. Data are representative of three biological replicates and shown as mean ± SD. *P* values were determined using a non-parametric one-way ANOVA (**P <* 0.05; ***P <* 0.01; ****P <* 0.001; *****P <* 0.0001; ns, not significant).

**Tables**

**Table S1 The accession numbers of 51 *tet*(X4)-positive plasmids from NCBI databases.**

| Serial number | Accession numbers | Plasmid types |
| --- | --- | --- |
| 1 | CP040909 | IncX1 |
| 2 | CP040929 |  |
| 3 | CP045998 |  |
| 4 | CP046002 |  |
| 5 | CP050037 |  |
| 6 | CP050046 |  |
| 7 | CP053047.1 |  |
| 8 | MN436006 |  |
| 9 | MN436007 |  |
| 10 | MT197111 |  |
| 11 | MT219821 |  |
| 12 | MT219822 |  |
| 13 | MT219825 |  |
| 14 | CP047572 | IncI |
| 15 | CP047578 |  |
| 16 | CP037909 | IncQ1 |
| 17 | MN848327 | IncFIB |
| 18 | MT219816 | IncA/C2 |
| 19 | CP037911 | Multiple |
| 20 | CP038140 |  |
| 21 | CP041443 |  |
| 22 | CP041443 |  |
| 23 | CP041449 |  |
| 24 | CP041453 |  |
| 25 | CP045188 |  |
| 26 | CP046004 |  |
| 27 | CP046007 |  |
| 28 | CP046717 |  |
| 29 | CP047460 |  |
| 30 | CP047466 |  |
| 31 | CP049354 |  |
| 32 | CP050041 |  |
| 33 | CP050174 |  |
| 34 | CP058949 |  |
| 35 | CP059044 |  |
| 36 | CP060586 |  |
| 37 | CP075463 |  |
| 38 | CP075467 |  |
| 39 | CP075470 |  |
| 40 | CP075471 |  |
| 41 | MK134376 |  |
| 42 | MN381965 |  |
| 43 | MN101858 |  |
| 44 | MN101856 |  |
| 45 | MT219817 |  |
| 46 | MT219818 |  |
| 47 | MT219819 |  |
| 48 | MT219820 |  |
| 49 | MT219823 |  |
| 50 | MT219824 |  |
| 51 | MT219826 |  |

**Table S2 Basic information of three *tet*(X4)-positive plasmids encoding H-NS protein.**

| Plasmids | Size (bp) | Conjugative | Inc-type | *hns* repeats | Organisms |
| --- | --- | --- | --- | --- | --- |
| pHS14-2 | 57k | + | IncX1 | one | *K. pneumoniae* |
| pRF14-1 | 50k | + |  | one | *E. coli* |
| pSC4R | 55k | + |  | one | *C. freundii* |

**Table S3 RT-qPCR primers used in this study.**

| Primers | Sequence (5' to 3') |
| --- | --- |
| *hns*-RNA-F | TGAAGAGCGCCGAGAAGAAG |
| *hns*-RNA-R | CCGCGACCAGACCAGTATTT |
| *tet*(X4)-RNA-F | CGATTGGGACGAACGCTACA |
| *tet*(X4)-RNA-R | TCCTGCAAAAGGAGGCATCA |
